# Development of Edible Films Based on Nostoc and Modified Native Potato Starch and Their Physical, Mechanical, Thermal, and Microscopic Characterization

**DOI:** 10.3390/polym16172396

**Published:** 2024-08-23

**Authors:** Antonieta Mojo-Quisani, Daniel A. Ccallo-Silva, David Choque-Quispe, Miriam Calla-Florez, Carlos A. Ligarda-Samanez, Raúl Comettant-Rabanal, Raul Mamani-Condori, Víctor J. Huamaní-Meléndez

**Affiliations:** 1 Agroindustrial Engineering, National University of San Antonio Abad del Cusco, Cusco 08000, Peru; 114516@unsaac.edu.pe (D.A.C.-S.); miriam.calla@unsaac.edu.pe (M.C.-F.); 173338@unsaac.edu.pe (R.M.-C.); 2 Agroindustrial Engineering, José María Arguedas National University, Andahuaylas 03701, Peru; caligarda@unajma.edu.pe; 3 Universidad Privada San Juan Bautista, Facultad de Ingenierías, Escuela Profesional de Ingeniería Agroindustrial, Carretera Panamericana Sur Ex km 300, La Angostura–Subtanjalla, Ica 11004, Peru; raul.comettant@upsjb.edu.pe; 4 Department of Food Engineering and Technology, São Paulo State University (UNESP), Campus of São José do Rio Preto, São Paulo 15385-000, Brazil; victor.melendez@unesp.br

**Keywords:** Andean potato starch, biodegradable coatings, biomass, cyanobattery-based films, *Nostoc sphaericum*

## Abstract

Considering the potential of biopolymers from underutilized Andean sources in Peru to improve the characteristics of edible films, this work aimed to evaluate the formation of a polymeric matrix composed of Nostoc and modified potato starch for the formulation of edible films for food coating. The effects of polymer matrix ratio and drying temperature on films obtained by thermoforming were studied, determining the water vapor permeability and mechanical properties using a multifactorial design. Additionally, thermal properties were characterized by TGA and DSC, and structural properties by FT-IR and scanning electron microscopy. The results showed that the films exhibited lower solubility, lighter hues, better water vapor resistance, higher tensile strength, and improved thermal stability with increasing modified starch content. The formulation with higher Nostoc content exhibited a more homogeneous surface according to microscopy images, and no new chemical bonds were formed by adding modified starch and Nostoc to the polymer matrix, according to FT-IR spectra. These findings are promising and suggest using Nostoc for elaborating edible films composed of native and modified starch from native Andean potatoes as bio-based materials with potential application in the food industry.

## 1. Introduction

Considering the relevance of synthetic plastic polymers in the preservation of perishable foods with high moisture and water activity, it is crucial to address the dilemma posed by their widespread use in the food industry [1,2]. Although synthetic, petroleum-based products have proven effective in preserving fruit and vegetables post-harvest, their detrimental environmental impact cannot be disregarded [3]. In this context, biodegradable films and coatings for food preservation are essential to reducing contamination and ensuring food safety, which is evolving towards a circular economy, prioritizing bioplastics to reduce the carbon footprint [1]. Transitioning to bio-alternative plastics is crucial for a sustainable and safe food supply system. Increasing interest in research and development of technologies to minimize food losses during storage, using biodegradable, healthy, and secure products [4], using bio-based packaging materials derived from various renewable resources, such as bioresources from microorganisms and microalgae [5]. Natural films and coatings from polysaccharides (cellulose, pectin, starch, alginates, chitosan, and gums) and vegetal or animal proteins (collagen, whey, zein, gluten, and glycine) offer a more sustainable and eco-friendly alternative [6,7]. With simple and easily scalable procedures involving green solvents or requiring little energy, these films can protect food against oxidation or growth of microorganisms [8].

Although edible films generally have many benefits, such as degradability, affordability, and ease of recycling, their technological use has limitations due to their mechanical and barrier properties, high water sensitivity, low surface functionality, or poor printability and adhesiveness [9]. To overcome these limitations, several researchers have developed and characterized starch-based coatings as a polymeric matrix [10], incorporating various biocomponents such as waxes, essential oils, proteins, and nanoparticles to create biodegradable hybrid packaging with reduced water vapor permeability, low gas transmission, antimicrobial activity, and improved mechanical properties [11,12,13].

The starch structure and composition vary according to botanical source and processing conditions; this plant-derived glucose polymer mainly comprises amylose and amylopectin. Its properties include biodegradability, abundance, and low cost for the creation of edible films [14]. Native starch is poorly soluble in room-temperature water due to its molecular structure, but when heating in water, the starch granule expands and triggers the gelatinization process, where the granules swell, the semi-crystalline structure collapses, and the molecules solubilize, forming a continuous film. However, during cooling, retrogradation occurs, and the gelatinized starch re-associates. This thermal process modifies the starch polymer, resulting in a film with limited mechanical characteristics and water permeability [15,16]. 

Among the methods of starch modification include chemical, enzymatic, physical, and genetic modifications [17]. Modified starch has attracted much academic and industrial attention due to the substantial improvement in the formation and properties of edible films [18]. The enzymatic treatment and retrogradation produces more compact amylose crystalline structures; the enzymes act on the α-(1,6)-glycosidic linkages of amylopectin, increasing the amylose content, which improves the mechanical properties of the film [17]. Films with more modified starch generally have high mechanical strength, water resistance, and better gas permeability [19]. It has been found that the mechanical properties of composite films are highly dependent on the interactions between the polymer matrix and the fillers; their molecular forces are strengthened when they bind to the hydroxyl groups and other possible hydrogen or van der Waals bonds of the starch macromolecules [20]. In this context, modified starch was used as a filler material in the polymeric matrix in this research. On the other hand, using plasticizers in the formulations allows for obtaining more stretchable films but with lower mechanical characteristics [21] and better transparency [22]. 

Marine algae are accessible and can form films with good mechanical and gas barrier properties due to their ability to establish chemical bonds and intermolecular forces that enhance integration with polymeric matrices [16,23]. Including polysaccharides extracted from algae and microorganisms in producing edible biopolymer films has opened up new possibilities in industries such as food, cosmetics, pharmaceuticals, and medicine [24,25]. Nostoc (*Nostoc sphaericum*), or cushuro in Peru, is a climate-resistant and underutilized Andean seaweed found in lakes and/or springs above 3000 m of altitude [26]. This microorganism is a freshwater cyanobacterium that forms spherical colonies composed of filaments or trichomes considered genetically safe for human consumption, containing highly photostable compounds that act as a photoprotectant and antioxidant [27]. For these functional characteristics, it is widely used in dietary supplements and therapeutic products because it has a high nutritional value [28,29]. However, it has some drawbacks in film formation, such as low tensile strength and water solubility; but such characteristics can be improved by adding other biopolymers, such as modified starches or natural active agents [30].

Some researchers have successfully modified the structure of starch-based edible films by adding co-biopolymers to improve mechanical properties in films [31,32]. On the other hand, few works, as reported by Rodriguez et al. [33], have used Nostoc sphaericum, where films were made with the polysaccharides extracted from Nostoc in the presence of glycerol, while Seguil et al. [34] studied the physicochemical qualities of strawberries coated with Nostoc-based edible films. 

Despite advances in research on biodegradable edible films, the combination of native potato starch modified with Nostoc for the development of edible films has not yet been comprehensively studied. This innovative combination has the potential to significantly improve both the mechanical and barrier properties of the films. Native potato starch provides a solid and flexible base, while Nostoc with its gelling and network-forming properties can increase tensile strength and durability. In addition, the integration of Nostoc is expected to strengthen barrier properties, reducing moisture and gas permeability, which could extend the shelf life of food products and offer a more efficient and sustainable alternative in the edible packaging industry. Therefore, this research work aimed to study the effect of the concentration of modified starch in a matrix composed of Nostoc and potato starch and to evaluate the influence of thermoforming temperature on the physical, mechanical, and structural properties of edible films.

## 2. Materials and Methods

### 2.1. Raw Materials and Reagents

A native potato variety named Yurac Anca was obtained from the Apachaco community, Coporaque district, Espinar province, Cusco, Peru (14°52′19.8″ S, 71°31′48.4″ W, and 4001 m altitude) from a 2021 harvest. Nostoc samples were collected from the spring of Chaupimayo hamlet, community of Concaja, district of Suykutambo, province of Espinar, Cusco, Peru in 2021 (14°56′43.7″ S, 71°37′55″ W, and 4210 m altitude). The chemical reagents used in the present study were α-amylase enzyme (IFCC Liquid Amylase, Spain), glycerol (Merk Spectrum Chemical 1012, New Brunswick, Canada) ACS reagent, and distilled water.

### 2.2. Extraction of Native Starch and Obtaining Modified Starch

Starch extraction was carried out using the wet method, which consisted of washing the potatoes with plenty of water and cutting them into uniform cubes of about 2 cm. They were then mashed in an industrial blender (Surco L-20, Lima, Peru) for 3 min at a potato/water ratio of 1:3. The mash was filtered through a polyester sieve with a pore size of about 0.05 mm to separate the starch-rich solution from the solid phase composed of fibers. The starch solution obtained was allowed to stand at room temperature for 2 h, and the water was drained. Then, it was washed with distilled water two more times and allowed to stand intermittently for 3 h to purify the starch. The wet starch was spread on a tray and dried at room temperature for 72 h. 

The modified starch was prepared by mixing 1 g of native starch and 100 mL of distilled water; then, 50 µL of α-amylase (A = buffer/enzyme and B = buffer/substrate) was added and stirred at 150 rpm on a magnetic stirrer (CAT, Model M6, Germany) for 4 h at 50 °C in an incubator (DIN 12880, BINDER, Tuttlingen, Germany). It was then cooled to room temperature and centrifuged at 4000 rpm for 10 min. The supernatant was removed, and the samples obtained were dried in a vacuum oven at 20 °C and 10 mBar pressure for 4 h.

### 2.3. Obtaining of Powdered Nostoc

The collected Nostoc samples were washed with distilled water and then sorted, discarding those with physical damage. The samples were then disinfected with 10 mL of sodium hypochlorite (The Clorox Company, Broadway, Oakland, CA, USA) dissolved in 10 L of water for 2 min, followed by a rinse with potable water and finally with distilled water. The samples were then ground in a blender in a 1:3 ratio of Nostoc and distilled water. They were then sieved using a sieve shaker (Retsch AS200, Haan, Germany) with a 63 mm mesh; the viscosity of the mixture was adjusted to 80.70 cP; and spray dehydration was performed using a mini spray dryer (Buchi B-290, Flawil, Switzerland) with an inlet temperature of 100 °C, an air velocity of 600 L/s, and an extraction rate of 38 m^3^/h. The resulting dehydrated powder was stored in lidded PET tubes for preservation and use.

### 2.4. Edible Film Preparation

Edible films were obtained based on the formulation of Choque et al. [35], with some modifications, using a mixture of Nostoc powder (PN) and potato starch (PS) as a polymeric matrix, maintaining a ratio of 1:30 (PN/PS) between these two biopolymers. Modified potato starch (MPS) was added as reinforcement and glycerol (G) as a plasticizer, and 2 drying temperatures were varied for 3 formulations (Table 1).

Suspensions of 2% polymer matrix (*w*/*v*) and MPS were subjected to gelatinization (60 °C) under continuous stirring for 20 min. Then, it was mixed in the order (PN/PS)–(MPS)–(G) according to the formulation (Table 1), with constant stirring at 600 rpm and 60 °C for 5 min. After complete and homogeneous dissolution, 50 mL of the filmogenic solution was poured into Petri dishes of 134.90 mm diameter (height 3.52 mm) for thermoforming. Each formulation was placed in a forced-convection dryer (Binder FED, Tuttlingen, Germany) at the temperature of each formulation (Table 1). They were then allowed to cool to room temperature to separate the films from the Petri dishes and conditioned in desiccators for storage and further characterization.

### 2.5. Thickness

A digimatic micrometer (Mitutoyo MDC-1 PX, Kamata, Japan) was used to measure the thickness. Three strips of film, measuring 1 × 3 cm, were measured and placed between two glass plates. The difference between the thickness measurements of the film strips was used to determine the thickness of the glass plates with and without the sample.

### 2.6. Color

The Konica Minolta colorimeter (CR-5, Tokyo, Japan) was used to determine the film sample color in CIELab spacewhere L*: lightness scale (0 = black and 100 = white), a*: red/green coordinates (positive values indicate red and negative values indicates green), and b*: yellow/blue coordinates (positive values indicate yellow and negative values indicates blue). For the chromatic color intensity (C*), hue angle (h*), and color difference (ΔE*), the following Equations (1)–(3) were used.

(1) $$C^{*}=\sqrt{a^{{*}^{2}}+b^{{*}^{2}}}$$

(2) $$h^{*}=arctan\frac{b^{*}}{a^{*}}$$

(3) $$\Delta E=\sqrt{{{(\Delta L}^{*})}^{2}+{{(\Delta a}^{*})}^{2}{{+(\Delta b}^{*})}^{2}}$$

### 2.7. Water Activity (a_w_)

Film samples of 1 × 1 cm were taken and placed in a desiccator for 24 h at 43% relative humidity (RH) and a temperature of 20–22 °C. The a_w_ of each sample was then measured with 5 replicates in a Rotronic (HygroPalm23-AW, Bassersdorf, Switzerland).

### 2.8. Transparency

To determine the transparency of the edible film, the sample was cut into rectangular pieces (8 × 33 mm) corresponding to the lateral area of the spectrophotometer cell and placed in the cuvette. The absorbance was measured at a wavelength of 600 nm using a spectrophotometer (UV–Vis Agilent Technologies i8, Santa Clara, CA, USA). 

### 2.9. Solvent Resistance

The plastic behavior of the films was tested with some solvents, acids, and bases according to the method described by Rodríguez et al. [36] with some modifications. Film samples of 0.5 × 1 cm were cut and placed in test tubes. Then, the films were immersed by adding 2 mL of a solution consisting of distilled water, 90% ethanol, hydrochloric acid (0.1 M), acetic acid (0.1 M), and sodium hydroxide (0.1 M), each at 20 °C for 10 h. The following criteria were used for the analysis: if no film particles are observed in the solvent, it is classified as highly soluble (HS); if small particles are observed in the film and no degradation occurs, it is classified as moderately soluble (MS); if the film hardly degrades, it is classified as low soluble (LS); and if the film remains intact in the solvent environment, it is classified as insoluble (NS).

### 2.10. Water Vapor Permeability (WVP)

The WVP was performed according to ASTME-96/E-96M-05 [37] with modifications made by Díaz [38] using test tubes of 13.63 mm in diameter to which 10 mL of distilled water was added and which were sealed with the edible film with a thickness of 0.153 mm. They were then placed in a rack and put in a desiccation hood with SiO_2_. The relative humidity inside the drying hood was 32% and was 50% inside the test tube at the same temperature, 16.8 °C. The samples were periodically weighed on an analytical balance (A&D HR-250AZ, Oxfordshire, UK) at a time frequency of 2 h for 34 h. The saturation pressure of the water vapor was 1934 Pa as a function of temperature. The water vapor transmission results were calculated by plotting the weight variation (Equation (4)) versus time, fitting the graph to a straight line, and using the slope of the line to calculate the water vapor transmission coefficient WVTC (Equation (5)), the water vapor transmission rate in the film WVTR (Equation (6)), and finally, the water vapor permeability equation WVP (Equation (7)):

(4) $${∆}_{w}=w_{i}-w_{n}$$

where Δ_w_: weight variation in the tube (g) in each period, w_i_: initial weight of the tube in the test, and w_n_: weight of the tube taken at a known time;

(5) $$WVTC=\frac{d_{m}}{d_{t}}\times \frac{1}{A}$$

where *dm*/*dt* are the mass difference over time, and A is the area of the exposed film;

(6) $$WVTR=\frac{WVTC}{P(R_{2}-R_{1)}}$$

P is the saturation vapor pressure at the experimental temperature (Pa), R_1_ is the relative humidity of the chamber in fraction, and R_2_ is the relative humidity inside the test tube in fraction.

(7) WVP=WVTR×Thickness

### 2.11. Solubility

Samples were prepared according to the method described by Escamilla et al. [39]; the films were cut into 2 × 2 cm rectangular pieces and weighed on an analytical balance (initial weight) (A&D Weighing, HR-250AZ, UK). Subsequently, they were immersed in 80 mL of distilled water in a 100 mL beaker at room temperature and transferred to a multiple magnetic stirrer (OXFORD, MHS-10L EE. UU); after filtering, the film was placed in a Petri dish and dried in an electric oven (Whirlpool, WOB60M Argentina) at 110 °C for 24 h until a constant weight was reached (final weight). Finally, the soluble material was calculated according to Equation (8):

(8) $$\%Solubility=\frac{W_{i}-W_{f}}{W_{i}}\times 100$$

where W_i_ is the initial weight, and W_f_ is the final weight. 

### 2.12. Thermogravimetric Analysis (TGA)

The thermal stability of the raw materials and films was determined by a TGA. The samples were loaded into alumina (Al_2_O_3_) crucibles and transferred to the instrument (TA Instruments, TGA550, New Castle, DE USA). The instrument was equipped with Trios V5.0.0.44616T software. The heating was programmed from 20 to 600 °C. The heating rate was 10 °C/min, and the nitrogen supply was 50 mL/min.

### 2.13. Differential Scanning Calorimetric

The thermal transition properties of the raw materials and films were analyzed using a differential scanning calorimetry analyzer (TA Instruments, model DSC2500 Waters TM, New Castle, DE, USA) under a nitrogen atmosphere (50 mL/min). The samples were sealed in an aluminum tray and scanned from 20 to 200 °C at a heating rate of 5 °C/min. The equipment was stabilized through a baseline run under analytical conditions for 1 h.

### 2.14. Fourier-Transform Infrared (FT-IR) Spectroscopy

Tablets pressed with 0.1% of the film in KBr potassium bromide (IR Grade, Darmstadt, Germany) were prepared and taken to the transmission module of a Thermo Fisher FT-IR (Fourier-transform IR spectroscopy) spectrometer (Nicolet 50, Waltham, MA, USA). Readings were made in wavenumber with a range of 4000 to 400 cm^−1^ and a resolution of 4 cm^−1^.

### 2.15. Scanning Electronic Microscopy (SEM)

A scanning electron microscope (SEM, Prism E, Thermo Fisher, MA, USA) with Oxford Inca 350 X-ray energy dispersive microanalysis (EDAX) was used. The films were placed on adhesive tape and brought into the vacuum chamber of the equipment at 30 kV acceleration and 1000× magnification. The equipment software Velox 2.9 (FEI, Waltham, MA, USA) identified the biopolymer surface.

### 2.16. Tensile Strength and Percentage of Elongation

The films were cut into strips (1 × 10 cm) and subjected to tensile forces using a Pasco machine (ME-8236, Roseville, CA, USA) based on the ASTM 882 [40] standard method (ASTM, 2001). Tensile strength (TS) and percent elongation at break (E %) were determined. The results are the average of five samples, where TS and E % at the fracture point of the film were calculated according to the following Equations (9) and (10) proposed by Kurt and Kahyaoglu [41]:

(9) $$TS=\frac{M}{T\times W}$$

where M is maximum force measurement (N), T is thickness (mm), and W is width of the film;

(10) $$E\;\%=\frac{d_{r}}{d_{0}}\times 100$$

where E % is the percentage of elongation after breakage, d_r_ is the breakage distance (mm), and d_o_ is the distance at the beginning of separation (mm).

### 2.17. Statistical Analysis 

A 3 × 2 factorial design was used, resulting in six treatments (Table 1) composed of two factors: formulation (3 levels) and drying temperature (2 levels). An analysis of variance (two-way ANOVA) and Tukey’s multiple comparison test at 95% confidence level were used to evaluate the response variables. Minitab v.20 test software was used to perform all statistical analyses, and data plots were created in Origin Pro version 2022.

## 3. Results

### 3.1. Thickness

**Figure 1:**
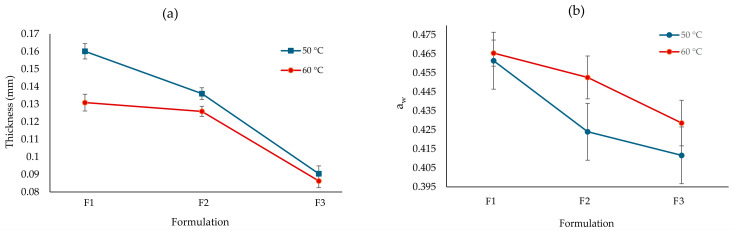
Factor interaction graph for thickness (**a**); factor interaction graph for a_w_ (**b**).

The results presented in Table 2 show the variability observed in the thicknesses of the edible films, which ranged from 0.090 to 0.160 mm, where the influence of the concentration of the filmogenic solution, the molecular weight of the biomolecules, and the geometry of the mold are observed [42]. F1 at 50 °C was the treatment that generated the highest thickness among all the samples (*p* < 0.05), followed by the same formulation at 60 °C. This shows the effect of the thermoforming temperature (Figure 1a), which shows that the higher the temperature, the lower the film thickness, as the temperature had a direct effect on the reduction in the viscosity of the filmogenic medium [43] (Table 2). F2 and F3 had lower thicknesses, possibly due to the lower content of Nostoc (polymeric matrix) and glycerol, the latter being a structuring material that favors the interaction between different polymers of anionic and cationic nature. These results confirm the finding of Kocira et al. [44], who report that plasticizers reduce the stiffness and increase the polymer extensibility by reducing the intermolecular forces acting between adjacent chains.

The notable differences between samples F1, F2, and F3, as well as the variations, were observed at different temperatures (60 and 50 °C); this shows the complexity inherent to the formation of these films. F1 at 50 °C was the film with the highest thickness, the one with the highest proportion of polymeric matrix, and the lowest amount of modified starch (Table 1); F3 at 60 °C was the film that showed the lowest thickness since it had the lowest proportion of modified starch. There was also a significant difference (*p* < 0.05) for the drying temperature, where the lower the temperature, the higher the film thickness.

The food-coating film thicknesses reported by several authors are within values found for starch/Nostoc, modified starch, and glycerol composite films (Table 2). Senturk et al. [45] reported values between 0.187 and 0.235 mm for alginate, glycerol, Tween 80, and sunflower oil films, while Arredondo et al. [46] obtained chitosan and sorbitol films with different thicknesses from 0.09 to 0.152 mm; similarly, Anchundia et al. [47] reported thickness from 0.11 to 0.17 mm, and Rocha et al. [48] found thicknesses from 0.10 to 0.18 mm. Kumari et al. [49] reported films based on fenugreek proteins with a thickness between 0.23 and 0.30 mm, values higher than those found in the present study. 

### 3.2. Water Activity (a_w_)

The a_w_ values ranged between 0.412 and 0.465 (Table 2), where it was observed that F2 and F3 at 50 °C had the lowest a_w_ values among all the samples (*p* < 0.05). This was due to the effect of the higher concentration of modified starch between 7 and 9%, which, having polymeric fragments of lower molecular weight with a higher number of hydrophilic groups, established a higher chemical interaction with the water molecules and a consequent reduction in the a_w_. This phenomenon, when the temperature was increased from 50 to 60 °C (Figure 1b), caused an increase in the activation energy and changes in the water adsorption properties, which led to an increase in the water activity of the films. The film formulation was a predominant factor in the determination of a_w_, and a direct relationship was observed between the content of powdered Nostoc in the polymeric matrix (PN/PS) and the a_w_ values; an inverse relationship was observed with the content of modified starch. This phenomenon was manifested in F1, which exhibited the highest a_w_ values. These results were lower than the values of the starch films developed by Radev and Pashova [50], which were between 0.5974 and 0.7882, and [35] with a_w_ values between 0.596 and 0.639 when using native potato starch and nopal mucilage. Similar results were found in edible jackfruit straw films with the addition of ginger extract, reported by Neswati et al. [51], and in edible films with the addition of corn husk cellulose nanocrystals [52]. Notably, all a_w_ values remained below the critical threshold of 0.6, considered the limit for microbial growth [53], and the recommended maximum value of 0.65 for starch-based foods [54]. This characteristic suggests a favorable microbiological stability for the developed films, which is crucial for their potential application in the food industry.

### 3.3. Transparency

Transparency is expressed as the ratio of absorbance to thickness (nm/mm). Therefore, high values indicate low transparency and high opacity [41]. The transparency of edible films was affected by the thermoforming temperature and film composition. The films processed at 60 °C were less transparent than those at 50 °C. This may be because the higher temperature favors the formation of a more compact crystalline structure in the film, which reduces light scattering. As for the film composition, it is observed that the F1 films are more transparent (low values) than the F2 and F3 films (Table 2). This may be because the F1 films had a higher amount of glycerol (Table 1), which caused the plasticizer to reduce the stiffness and increase the extensibility of the polymers. On the other hand, they contained a lower concentration of MPS polymeric material (Table 1), leaving voids in the film. While in F2 and F3 films, increasing the modified starch content and decreasing glycerol produced less transparent films. The same effect on transparency was found by Kurt and Kahyaoglu [41], whose values ranged from 3.74 to 4.30 for glucomannan and guar gum films with glycerin as a plasticizer. Meanwhile, Singh et al. [55] found lower transparency values of 1.02 for corn starch films and 0.54 for potato starch reinforced with chitosan and sorbitol as plasticizers.

### 3.4. Solubility

The films prepared at 50 °C are more water soluble than those prepared at 60 °C (Table 2). This may be because the lower temperature favors the formation of a more open structure in the film, which allows water to penetrate easily into the film. Regarding the film composition, it is observed that the F3 film is less soluble in water than the F1 and F2 films. This may be because the F3 film contains a higher amount of modified starch, which acts as a filler on the surface and creates a barrier effect, resulting in lower solubility and higher film resistance in polar environments. On the other hand, F1 and F2 films contain a higher amount of polymeric matrix (starch/Nostoc), which are hydrophilic materials and have higher glycerol content, whose plasticizing effect can interact with water and alter the film network through hydrogen bonds, reducing the cohesion between the starch/Nostoc polymeric matrix and increasing water solubility.

The solubility values of the PS–PN–MSP-based films were similar to those of Dick et al. [56], which were obtained between 52 and 84% solubility for films with chia mucilage, and González et al. [57], between 81 and 91% in edible films based on organic mucilage of opuntia ficus–indica Mexicana. However, they were higher compared to what was reported by Silva et al. [58] in films based on native cassava starch, where this parameter ranged between 10 and 23%. Galus and Kadzińska [59] found lower solubility values between 37 and 42% for whey-protein-based edible films with a transparency of 1.02 for corn starch films and 0.54 for potato starch reinforced with chitosan and sorbitol as plasticizers

### 3.5. Water Vapor Permeability (WVP)

The samples showed WVP values ranging from 1.26 × 10^−2^ to 7.55 × 10^−3^ g·mm/h·m^2^Pa (Table 2). Generally, the films dried at 60 °C showed a lower WVP than those dried at 50 °C. This is attributed to the formation of a denser and more compact structure in the films dried at higher temperatures, which reduces the permeability to water vapor because heat favors the compaction of polymers and the elimination of free spaces in the film structure. As for the formulations, F2 presented the lowest WVP at both drying times. It is presumed that F2 (7% MPS) can interact with the other components of the film, forming a more compact network that is resistant to the passage of water vapor. On the other hand, the addition of glycerol in the formulation increased the WVP; glycerol, being a small molecule, can penetrate the intermolecular matrix and facilitate the diffusion of water molecules, providing a mechanistic basis for understanding the increased permeability according to Dick et al. [56], which is confirmed in F1. The effect of modified starch and Nostoc (polymeric matrix) on the WVP of the films demonstrates the complexity of interactions in these systems; therefore, it is important to balance the composition of edible films to achieve the desired barrier properties. 

The water vapor permeability means of edible F1, F2, and F3 films show statistically significant differences (*p* < 0.05). F1 (92% PN/PS, 5% MPS, 3% G) showed the highest water vapor permeability, significantly different from F2 and F3. This can be attributed to its higher content of the polymeric matrix PN/PS (92%) and lower content of modified starch (5%). F2 (91% PN/PS, 7% MPS, 2% G) showed intermediate water vapor permeability, which is not significantly different from F3 but substantially different from F1. The slight increase in the modified starch content (7%) and the reduction in glycerol (2%) compared to F1 could contribute to a less porous structure. F3 (90% PN/PS, 9% MPS, 1% G) showed the lowest water vapor permeability, although not significantly different from F2. This formulation has the highest modified starch content (9%) and the lowest glycerol content (1%). The increased proportion of modified starch could contribute to a more compact and less porous structure. The observed trend suggests that the increased proportion of modified starch (MPS) and the decrease in glycerol (G) content are associated with reduced water vapor permeability. The properties of modified starch could explain this to form more cohesive films with better barrier properties. It is important to note that the effect of drying temperature (50 °C vs. 60 °C) was not statistically significant on water vapor permeability, indicating that the observed differences are mainly due to the composition of the formulations.

These findings suggest exciting possibilities for future research in the fields of food packaging and pharmaceuticals. They indicate that manipulation of PN/PS, MPS, and G ratios in formulations allows for the modulation of the water vapor barrier properties of edible films, which is crucial for their application in these fields.

In comparison with other studies, differences in permeability values were observed. For example, the lowest values were found in films based on cassava starch (3.74 − 6.84 × 10^−4^ g·mm/h·m^2^Pa), chia mucilage (1.31 − 4.42 × 10^−4^ g·mm/h·m^2^Pa), and edible films of cassava starch (3.02 − 4.75 × 10^−4^ g·mm/h·m^2^Pa) [56,60,61]. The results were similar to those reported by Barandiaran et al. [62], who obtained similar WVP values of 1.25 a 2.42 × 10^−3^ g·mm/h·m^2^Pa, using starch isolated from different varieties of Colombian potato, and in chitosan films (1.32 × 10 a 2.08 × 10^−2^ g·mm/h·m^2^Pa) and films based on cassava starch modified by corona treatment (1.58 × 10^−2^ g·mm/h·m^2^Pa) [9,63]. These values indicate that our results are within the range of permeability observed in the literature, suggesting a consistency in the ability of edible films to regulate the passage of water vapor. 

**Figure 2:**
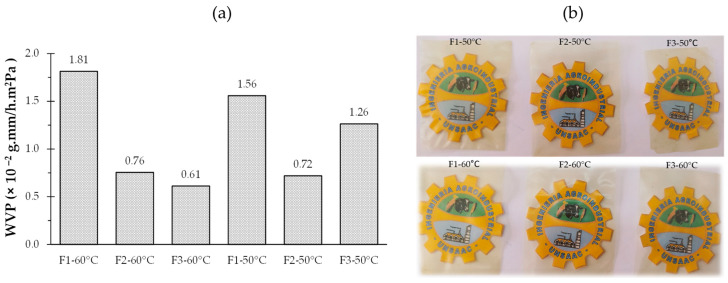
Water vapor permeability (**a**); edible films (**b**).

Figure 2a illustrates the water vapor permeability of edible films under different formulation conditions (F1, F2, F3) and drying temperatures (50 °C and 60 °C). A general trend of increasing permeability is observed as one moves from formulation F1 to F3, regardless of temperature. Films dried at 50 °C consistently exhibit higher permeability values than their counterparts at 60 °C for each formulation. Notably, formulation F3 at 50 °C exhibits the highest permeability (1.26 × 10^−2^), while F3 at 60 °C exhibits the lowest value (6.13 × 10^−3^). This variation suggests that both formulation composition and drying temperature significantly influence the water vapor barrier properties of the films, with a complex interaction between the two factors determining the final performance of the material in terms of permeability.

In the edible films (Figure 2b), variations are observed that could be attributed to modifications in the proportions of their components. F1, with higher polymer matrix (PN/PS) and glycerin content, exhibited a slightly opaquer appearance than the others. As the percentage of modified potato starch (MPS) increases and the concentration of glycerin decreases in F2 and F3, a gradual increase in the transparency of the films is observed. This phenomenon could be explained by the molecular interaction between the modified starch and the polymeric matrix, potentially leading to a more homogeneous structure and, thus, to a higher light transmission. Additionally, the glycerin content, known for its plasticizing capacity, could contribute to a more compact and less light-scattering matrix (F1); the SEM images corroborate these results.

### 3.6. Color

The color of edible films is an important property affecting their acceptability to the consumer. Table 3 shows the statistical analysis of the coordinates a*, b*, luminosity L*, hue h*, and chroma C* for the components and for the edible films, where no significant differences (*p* > 0.05) were found in the coordinates L*, a*, and b* and for the color attributes h*, C*, and ΔE*, which are not affected by the drying temperature or the film formulation (Table 3). It was also observed that the lightness was ~95 with a tendency to white, possibly due to the effect of the addition of glycerin and water in the formulation. The addition of modified starch increased the L* and a* values in the films; the opposite was reported by Jakubowska et al. [64], who developed and characterized active films of chitosan plasticizer, finding decreased L* and a∗ values for all films after reinforcement with quercetin, which caused the formation of darker films. On the other hand, Zhelyazkov et al. [65] reported L* values between 86.78 and 93.12, C* of 0.94 to 7.22, and h* from 86.57 to 221.86 for edible films loaded with potato starch and clove essential oil. In the present study, more constant values were found for the edible films than those reported by previous authors (Table 3).

### 3.7. Solvent Resistance

Table 4 provides information on the resistance in different solvent media. The obtained films were insoluble in alcohol and distilled water (Table 4), showing low affinity in polar solutions, which is favorable as a material with excellent water barrier properties. F3—60 °C—showed the highest water solubility of all the developed films, making it the best prototype as an edible coating material. However, the film’s resistance to solvents and pH suggests that starch combination with Nostoc polymers and glycerol possibly formed resistant complexes similar to resistant starch. Therefore, these films may act as insoluble fibers and potential probiotics. On the other hand, it is shown how solubility can vary not only with the formulation change but also with the drying temperature. In the case of NaOH (0.05 M), low to medium solubilization was observed, and the influence of drying temperature was reflected. Hydrochloric acid HCL (0.1 M) affected the film components, showing low to medium solubility with partial decomposition or swelling. Trichloroacetic acid CH_3_COOH (0.1 M) showed a solubility varying from low to medium, attributed to the film formulation.

### 3.8. Thermogravimetric Analysis (TGA)

**Figure 3:**
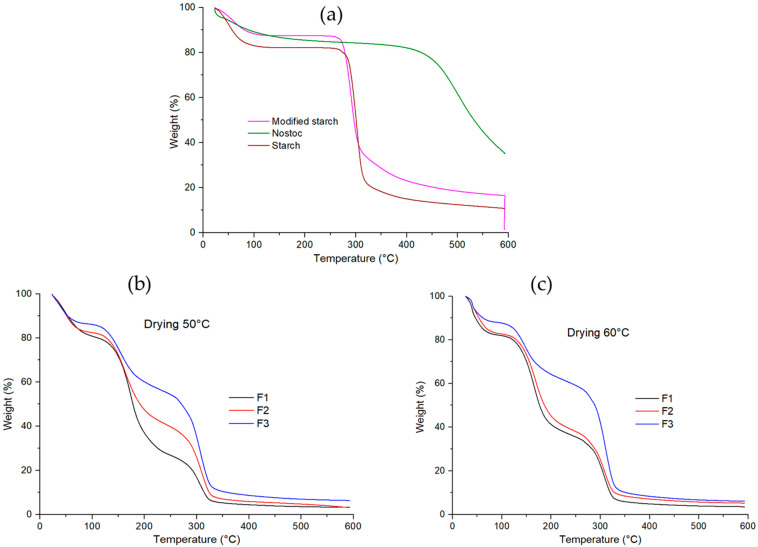
Thermogravimetric analysis (TGA) for raw materials and edible films.

The evaluation of their thermal properties makes it possible to determine the functions of the main components and individual additives, as well as some relationships between structures [66]. Both Nostoc and starch at high temperatures (>60 °C) are hydrophilic polymers with -OH groups, so they show thermal degradation, which can be similar to each other (Figure 3a). The four stages of weight loss are shown in Figure 3. In the initial stage (20–99.9 °C), the weight loss is between 18.86 and 12.9%, and this decrease is due to the increase in modified starch in F3 (Table 5). This is related to the evaporation of the weakly bound free water [67,68,69]; according to Choque et al. [52], this behavior is attributed to the large number of active sites with -OH groups that they possess, which gives them a high hygroscopic capacity. In the second exothermic stage, which occurs between 99.5 and 199.5 °C, weight loss increases rapidly by 40.41 to 22.30%. This is due to the loss of water and glycerin bound because the films were influenced by the higher polymeric matrix and glycerin content in the formulation [70] (Table 1), showing a decrease in weight loss from F1 to F3. According to Liu et al. [67], the loss corresponds to glycerol (60–120 °C); for Choque et al. [52], this stage corresponds to the combustion of glycerin at 220 °C. In the third stage (200–329.97 °C), a maximum mass loss rate between 31.44 and 52.41% is observed for all films, due to the degradation of macromolecules and polysaccharides [52,67,71]. The higher content of modified starch in the formulation increased the weight loss, suggesting that degradation of linear starch chains occurs at this stage. Figure 3b illustrates the weight loss patterns between thermoformed formulations at 50 °C, where F3 had the highest weight loss between 200 and 300 °C temperature compared to F1 and F2. This is possibly due to the effect of the lower glycerol content in the formulation which caused less plasticization of the water molecules allowing the water release at higher temperatures. While the influence of the temperature increases to 60 °C caused higher film weight loss (Figure 3c). In the fourth stage (330–591.15 °C), the weight change stabilizes as the material begins to carbonize. At this stage, the total decomposition of the residues refers to the carbon generated above 320 °C [52].

From these TGA results (Table 5), the maximum weight loss in % was for F1 (96.38–96.81%) > F2 (94.89–96.34%) > F3 (94.41–93.55%), respectively, indicating that the addition of modified starch (Table 1) improved the thermal stability of the F3 film. This finding may be due to the greater interaction between its components, having, at the same time, a greater compatibility and forming a more stable structure [70].

The TGA of the formulation components reveals that native potato starch undergoes three stages of weight loss. The first stage, between 20 and 232.49 °C, is due to water evaporation, accounting for 17.86% of the total weight loss. The second stage, from 232 to 339.59 °C, involves the decomposition of carbohydrates and low molecular weight peptides, with a 62.84% weight loss. The third stage, from 339.5 to 590.91 °C, corresponds to the degradation of high-molecular-weight polysaccharides, with an 10.80% weight loss and a maximum loss of 91.5%. On the other hand, Nostoc and modified starch also show three similar weight loss stages, with different temperature ranges and weight loss percentages in each stage (Table 5).

### 3.9. Differential Scanning Calorimetry (DSC) for Raw Materials and Edible Films

The F1 film with higher Nostoc content in the polymer matrix (PS/NP) and higher glycerol content showed higher Tg (Table 6). This may be due to the highly hygroscopic nature of glycerol and Nostoc, which tend to retain water in the film matrix. In this regard, Sanyang et al. [72] reported that higher glycerol concentrations produce more hydrophilic hydroxyl groups with active sites that water molecules can occupy. The addition of mucilage to the solution reduces the decomposition, making the films thermally more stable [73], which would be confirmed in F1 with higher Nostoc content in the matrix (Nostoc/starch); the Nostoc would have occupied the spaces between starch molecules in the polymeric matrix, improving the thermal stability of the films. Choque et al. [52] reported that the addition of nanocrystals reduces Tg and Tm when they are located in the interstitial spaces, accumulate as fillers, and act as thermal conductors; the same behavior was also found in this study (Table 7); as the nanocrystal content in the film increased, Tg decreased. The opposite was found when nanomaterials were introduced into biocomposite films [74].

**Figure 4:**
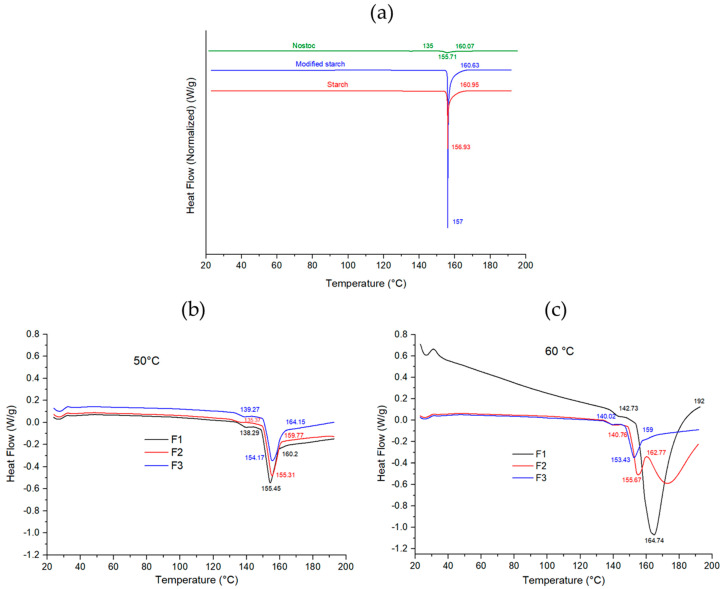
Differential scanning calorimetric (DSC) thermogram, (**a**): raw materials, (**b**): films at 50° C, (**c**): films at 60 °C.

The DSC thermograms of the films are shown in Figure 4. The endothermic peak in the range of 153 to 164 °C corresponds to the melting temperature of the film. On the other hand, film F1 showed an endothermic peak of higher intensity, whereas, in films F2 and F3, the intensity of the endothermic peak decreased slightly after the addition of the modified starch (Table 2) at the two drying temperatures, suggesting that the modified starch improved the thermal stability. As indicated by Biswas et al. [75], the incorporation of ZnO nanoparticles into the polymer matrix improved the thermal stability of the biocomposite films. Figure 4 shows the endothermic melting process for the film components, with sharp peaks for starch and modified starch as they are crystalline polymers, and the peak of the powdered Nostoc is imperceptible, probably because it is an amorphous polymer.

In the present study, we found a glass transition temperature (Tg) between 136 and 142 °C, values lower than those found by Sadaf [76], which ranged from 112 to 114 °C in an antimicrobial edible film made from basil seed mucilage (*Ocimum basilicum* L.) and sodium alginate. Also, higher endothermic melting temperature (Tm) values have been observed in several studies by Giannakas et al. [77], where they investigated edible films with novel hybrid nanostructures and observed sharp exothermic peaks around 185 and 230 °C. Charles et al. [78] studied films with a potato peel starch matrix plasticized with glycerol, showing endothermic peaks with Tm above 100 and up to 220 °C. Luo et al. [79] found melting temperatures around 179 °C in edible corn starch/bean protein films loaded with d-limonene particles. Similar results to ours were obtained by Choque et al. [52] in an edible film with the addition of corn husk cellulose nanocrystals, values between 150 and 156 °C, with high-intensity endothermic peaks. In contrast, Kumari et al. [49] obtained lower values, between 142 and 149 °C, in fenugreek films with pH between 9 and 12, respectively, while Li et al. [80] reported Tm from 138 to 148 °C in edible films of gelatin and sodium alginate cross-linked by pullulan.

### 3.10. Fourier-Transform Infrared (FT-IR) Spectroscopy

**Figure 5:**
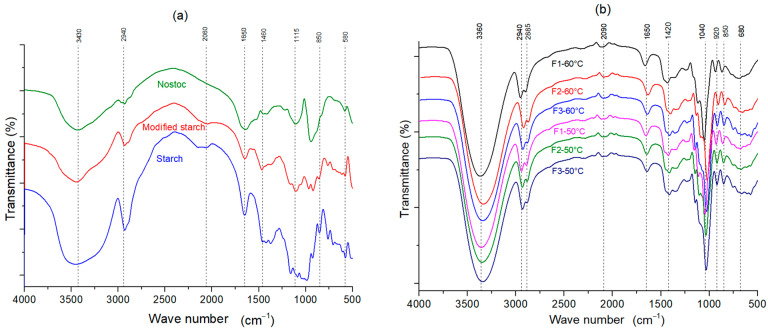
Fourier-transform infrared spectra corresponding to the components (**a**) and edible films (**b**).

The FTIR analysis revealed differences in the stretching vibration, absorption peak, and infrared spectra between the edible films and their components (Figure 5). The absorption peak of the starting material at 3430 cm^−1^ corresponds to the stretching vibration of NH and OH; in the composite film, a more robust and broader peak shift appears at 3360 cm^−1^, which would indicate the presence of alcohol. The peak at 2940 cm^−1^ corresponds to the CH vibration of the methyl group of the ester, which is more robust for native starch, which remains in the film with a higher peak intensity because the film contains a higher concentration of native starch. For modified starch and Nostoc, this is attributed to the CH-stretching vibration of the methyl groups present in its structure (Figure 5). At about 1650 cm^−1^, native starch, powdered Nostoc, modified starch, and all films showed stronger C=C-stretching vibrations. On the other hand, C–O, C–H, and –OH stretches occur around 1460 cm^−1^, corresponding mainly to native starch with the higher strength of the polymerized carbohydrate chains remaining in the film.

When comparing the spectra of all films, no new peaks were found, which means that no new chemical bonds were formed due to the addition of modified starch and Nostoc in the polymer matrix. All the spectra of the composite films presented distinctive peaks in the region from 3360 to 680 cm^−1^. The broad bands around 3360 cm^−1^ in all the samples indicate the presence of hydroxyl (–OH) groups with intermolecular hydrogen-stretching bonds, characteristic of polysaccharides. The peak around 2940 cm^−1^ probably corresponds to the stretching vibration of a CH alkane group within the biopolymer chain [73]. Representative peaks of the protein structures were identified at 1650 and 1420 cm ^−1^. The C–H- and C–O–H-bending vibrations of the carboxylic acid could be associated with the band around 1420 cm ^−1^, while the C–O–H- and C–O–C-stretching vibrations, responsible for the peak observed at 1040 cm^−1^, were identified at 1650 and 1420 cm^−1^ [74,75], which were present in all films. These characteristics are attributed to the contribution of the hydroxyl groups of glycerol, promoting dipole–dipole-type hydrogen bond interactions between starch, Nostoc, and glycerol, demonstrating their hydrophilic nature [35].

The bands in the FTIR spectra at approximately 1047 cm^−1^ and 1022 cm^−1^ are susceptible to changes in crystallinity. The band at 1047 cm^−1^ is associated with ordered or crystalline starch, while the band at 1022 cm^−1^ is characteristic of amorphous starch [81,82,83]. The ratio of the heights of these bands can express the amount of ordered starch to amorphous starch, providing information on the crystallinity of the films. The approximate absorption bands at 994 cm^−1^ are sensitive to changes in crystallinity and water content, reflecting intramolecular hydrogen bonding within the starch structure [81]. These bands can be distinguished in the spectrum corresponding to native and modified starch. However, due to the overlapping of several stretching bands in this region, they cannot be distinguished in the film spectra.

The ratio of absorption bands 1047:1022 turned out to be relatively high, indicating a high degree of molecular organization, resulting in 1.00 for native starch and 0.94 for modified starch, confirming the high degree of crystallinity of native starches from tubers, coinciding with the values of 1.112 for native starch and 1.083–0.947 for modified starch, as reported by Pourmohammadi et.al. [84].

### 3.11. Scanning Electron Microscopy (SEM) of the Best Edible Films

**Figure 6:**
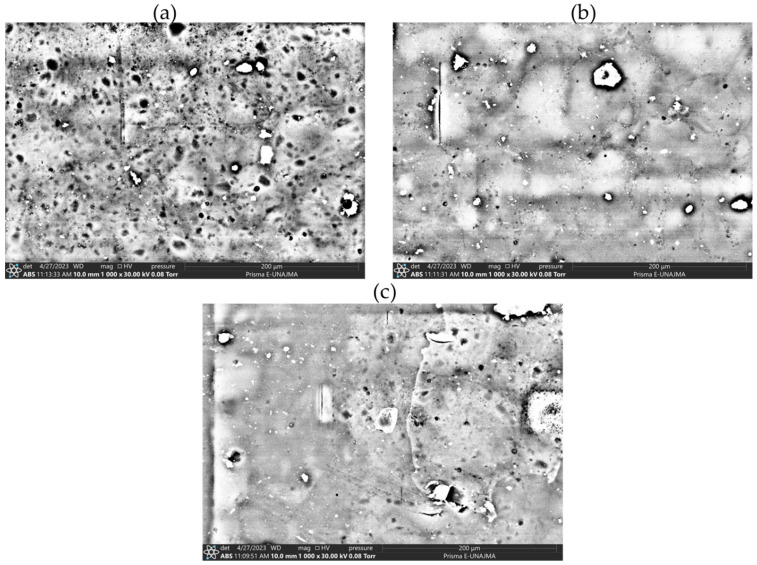
Scanning electron microscopy (SEM) of edible films (60 °C). (**a**) F1 (PN/PS: 92%, MPS: 5%, G: 3%); (**b**) F2 (PN/PS: 91%, MPS: 7%, G: 2%); and (**c**) F3 (PN/PS: 90%, MPS: 9%, G: 1%).

The microstructural characterization by SEM and the results of mechanical and barrier properties of the edible films reveal a significant correlation between composition, morphology, and functional performance. Formulations F2 and F3, with lower polymeric matrix content (Table 1), exhibited a more homogeneous and compact microstructure attributable to the incorporation of modified starch (Figure 6). This component acted as an effective filler, dispersing uniformly in the polymeric matrix and occupying the interstitial voids, resulting in a more uniform surface than F1. However, the SEM micrograph reveals that certain irregularities and pores persist, which could influence the mechanical and barrier properties. The compatibility between Nostoc and modified potato starch is moderate, a crucial factor for the effectiveness and functionality of the edible material. The structural configuration of F2 and F3 resulted in a lower water vapor permeability (WVP), suggesting that the modified starch facilitated the formation of a denser polymeric network resistant to the diffusion of water molecules. In contrast, formulation F1, characterized by a higher proportion of glycerol, showed a more heterogeneous surface with abundant voids, correlating with an increase in WVP due to glycerol’s ability to facilitate molecular mobility and water diffusion. Additionally, F1 exhibited lower mechanical strength and elongation (Table 7), attributable to the glycerol-induced decrease in intermolecular cohesion.

The presence of fractures and other defects observed suggests the need to optimize the film formation process to improve its uniformity and strength. These findings underscore the critical importance of composition and processing in modulating the microstructure and, consequently, the functional properties of edible films, opening avenues for future improvements in formulation and processing techniques.

### 3.12. Tensile Strength and Percentage of Elongation

Tensile strength is the capacity of a material to resist rupture under tension (MPa), while elongation at break is the elongation before rupture expressed as a percentage [85]. The mechanical properties, such as tensile strength (TS), Young’s modulus (YM), and elongation (E %) of the formulations (Table 7), showed that the incorporation of modified starch in the film formulation caused a significant (*p* < 0.05) increase in tensile strength from 0.31 MPa to 4.10 MPa (60 °C) and from 0.35 to 3.02 MPa (50 °C). Similar findings were found by Farajpour et al. [86], who found an increase in the density of the films with the addition of nanoparticles, which improved the tensile strength. The results obtained in the present study were similar to those of Umeohia and Olapade [87]. Such values ranged from 0.289 to 6.374 N/mm^2^ in edible film made from tomato skin fiber and moringa, and Zhou et al. [88] found values between 0.80 and 2.08 MPa in cassava starch and cinnamon essential oil films. Higher values were obtained by Farajpour et al. [86] between 10 and 22 MPa in edible films of potato starch and olive oil reinforced with zein nanoparticles, while Lopez-Polo et al. [89] reported values between 7.9 and 37.8 MPa in films produced with hydroxypropyl methylcellulose.

On the other hand, the addition of glycerol to the films between 1, 2, and 3% decreased the values of % elongation at break. It has been observed that, with the decrease in the plasticizer concentration in the film formation solution, the tensile strength and elongation increase due to the modified starch, which decreased the intermolecular spaces in the film, and the glycerol molecules decreased their cohesion with the starch and their network strength; consequently, the elongation of the film decreased (Table 7). A similar behavior was observed by López-Polo et al. [89] and Das et al. [85]: that a higher concentration of glycerol in the film formulation caused a decrease in the tensile strength and Young’s modulus values.

## 4. Conclusions

Factors such as the formulation of the polymer matrix and the drying temperature influenced the characteristics of the films. A higher content of modified starch favored greater thickness, while a lower content of this component, and a lower amount of glycerol promoted greater transparency. On the other hand, the presence of modified starch and glycerol affected the solubility in aqueous solutions, which affected the film strength. Likewise, the presence of glycerol and modified starch influenced the luminosity and tonality, generating lighter shades. It was observed that the addition of modified starch can improve the resistance to the passage of water vapor in the films, while glycerol can increase their permeability by facilitating the diffusion of water molecules. The formulation affected the mechanical properties; a significant increase in the tensile strength of the films was observed when incorporating modified starch, with values of 4.10 MPa at 60 °C and 3.02 MPa at 50 °C. On the other hand, the glycerol addition decreased the elongation percentage of the films. 

The addition of modified starch in the films composed of Nostoc, starch, and glycerol improved their thermal stability, which could be attributed to greater interaction and compatibility among the components, forming a more stable structure in the F3 formulation, which had the higher modified starch content. Infrared spectra revealed that no new chemical bonds were formed upon adding modified starch and Nostoc to the polymeric matrix of the films. Still, changes were observed in the vibrations and intensities of peaks characteristic of functional groups, indicating hydrogen-bonding interactions between the components. Finally, microscopy analysis showed that formulation 3 with higher Nostoc content presented a more homogeneous surface with fewer voids, suggesting that the Nostoc contributed to better dispersion and packing of the modified starch granules within the polymeric matrix.

## Figures and Tables

**Table 1:** Edible film formulations composed of a polymer matrix in different proportions.

Treatment	Temperature (°C)	Formulation	Components		
			PN/PS (%)	MPS (%)	G (%)
T1	60	F1	92	5	3
T2	60	F2	91	7	2
T3	60	F3	90	9	1
T4	50	F1	92	5	3
T5	50	F2	91	7	2
T6	50	F3	90	9	1

Native potato starch (PS), Nostoc powder (PN), modified potato starch (MPS), and glycerin (G).

**Table 2:** Physical properties of thickness, water activity (a_w_), transparency, solubility, and water vapor permissibility (WVP) in edible films.

Edible Film	Thickness (mm)	a_w_	Transparency	Solubility (%)	WVP (g·mm/h·m^2^Pa)
	$\bar{x}\pm$SD	$\bar{x}\pm$SD	$\bar{x}\pm$SD	$\bar{x}\pm$SD	$\bar{x}$ ± SD
F1-60 °C	0.131 ± 0.005 ^bc^	0.465 ± 0.007 ^a^	2.612 ± 0.071 ^d^	62.238 ± 5.743 ^abc^	1.81 × 10^−2^ ± 0.01 ^a^
F2-60 °C	0.123 ± 0.003 ^c^	0.453 ± 0.011 ^a^	4.567 ± 0.027 ^c^	64.408 ± 0.191 ^ab^	7.55 × 10^−3^ ± 0.00 ^b^
F3-60 °C	0.086 ± 0.004 ^d^	0.429 ± 0.012 ^b^	5.773 ± 0.147 ^a^	45.639 ± 3.939 ^c^	6.13 × 10^−3^ ± 0.00 ^b^
F1-50 °C	0.160 ± 0.004 ^a^	0.461 ± 0.007 ^a^	1.695 ± 0.037 ^f^	80.232 ± 2.629 ^a^	1.56 × 10^−2^ ± 0.00 ^ab^
F2-50 °C	0.136 ± 0.003 ^b^	0.424 ± 0.009 ^c^	4.765 ± 0.032 ^b^	56.635 ± 8.517 ^bc^	7.19 × 10^−3^ ± 0.00 ^b^
F3-50 °C	0.090 ± 0.004 ^d^	0.412 ± 0.014 ^c^	2.151 ± 0.003 ^d^	57.504 ± 6.320 ^bc^	1.26 × 10^−2^ ± 0.00 ^ab^

Results expressed as mean ± SD. Different letters in the same column indicate significant differences between treatments using Tukey’s test (*p* < 0.05).

**Table 3:** Color parameters in CIElab space of edible films composed of a native starch/Nostoc polymeric matrix with modified starch.

Samples	L*	a*	b*	h*	C*	ΔE*
	$\bar{x}$ ± SD	$\bar{x}$ ± SD	$\bar{x}$ ± SD	$\bar{x}$ ± SD	$\bar{x}$ ± SD	$\bar{x}$ ± SD
F1 (60 °C)	94.97 ± 0.01 ^a^	−1.51 ± 0.01 ^a^	9.33 ± 0.01 ^a^	99.32 ± 0.08 ^a^	8.07 ± 0.01 ^a^	7.97 ± 0.01 ^a^
F2 (60 °C)	95.08 ± 0.00 ^a^	−1.01 ± 0.00 ^a^	7.34 ± 0.00 ^a^	99.85 ± 0.01 ^a^	6.59 ± 0.01 ^a^	6.49 ± 0.01 ^a^
F3 (60 °C)	94.90 ± 0.00 ^a^	−1.09 ± 0.00 ^a^	7.49 ± 0.01 ^a^	98.08 ± 0.06 ^a^	9.48 ± 0.01 ^a^	9.39 ± 0.01 ^a^
F1 (50 °C)	94.97 ± 0.00 ^a^	−1.31 ± 0.01 ^a^	7.97 ± 0.01 ^a^	99.19 ± 0.06 ^a^	9.46 ± 0.01 ^a^	10.72 ± 0.01 ^a^
F2 (50 °C)	95.60 ± 0.00 ^a^	−1.14 ± 0.00 ^a^	6.50 ± 0.01 ^a^	97.84 ± 0.00 ^a^	7.41 ± 0.00 ^a^	7.34 ± 0.00 ^a^
F3 (50 °C)	95.43 ± 0.00 ^a^	−1.33 ± 0.01 ^a^	9.39 ± 0.01 ^a^	98.28 ± 0.01 ^a^	7.57 ± 0.01 ^a^	7.49 ± 0.01 ^a^
Starch	90.73 ± 0.09	0.23 ± 0.02	3.60 ± 0.03	86.35 ± 0.28	3.61 ± 0.02	-
Modified starch	88.22 ± 0.05	0.33 ± 0.01	2.71 ± 0.01	82.98 ± 0.09	2.73 ± 0.01	-
Pulverized Nostoc	50.44 ± 1.34	7.20 ± 0.81	37.22 ± 0.85	100.95 ± 1.19	37.92 ± 0.87	-

Results expressed as mean ± SD. Different letters in the same column indicate significant differences between treatments using Tukey’s test (*p* < 0.05). L* (0 = black and 100 = white), −a* (green), a* (red), −b* (blue), b* (yellow), h* (hue angle), and chroma color intensity (C*); ΔE*: color variation.

**Table 4:** Solvent resistance of edible films.

Formulation	Alcohol 90°	NaOH (0.05 M)	HCL (0.1 M)	Distilled Water	CH_3_COOH (0.1 M)
F1-60 °C	I	LS	MS	I	LS
F2-60 °C	I	LS	LS	I	MS
F3-60 °C	I	LS	LS	LS	MS
F1-50 °C	I	LS	LS	I	LS
F2-50 °C	I	MS	MS	I	MS
F3-50 °C	I	LS	MS	I	MS

I: insoluble, LS: low solubility, MS: medium solubility, CH_3_COOH: trichloroacetic acid.

**Table 5:** Weight loss (%) and degradation temperature by stages for edible components and films.

Material	First Stage		Second Stage		Third Stage		Fourth Stage		Weight Loss (%)
	$\bar{x}$ ± SD	T (°C)	$\bar{x}$ ± SD	T (°C)	$\bar{x}$ ± SD	T (°C)	$\bar{x}$ ± SD	T (°C)	$\bar{x}$ ± SD
Pulverized Nostoc	12.56	85.31	61.90	347.02	10.93	463.05	-	-	85.39
Starch	17.86 ± 0.02	232.49	62.84 ± 0.15	339.59	10.80 ± 3.50	590.91	-	-	91.50 ± 1.34
Modified starch	12.81 ± 0.26	240.03	59.17 ± 2.86	340.00	12.85 ± 1.21	593.00	-	-	84.83 ± 1.44
F1 (60 °C)	18.29 ± 0.31	81.72	40.41 ± 0.42	199.99	34.16 ± 0.15	329.97	3.53 ± 0.05	591.15	96.38 ± 0.01
F2 (60 °C)	17.64 ± 0.30	99.95	38.31 ± 0.19	199.97	34.62 ± 0.22	329.96	4.36 ± 0.10	590.21	94.89 ± 0.17
F3 (60 °C)	12.90 ± 0.64	99.03	22.30 ± 1.97	199.59	52.41 ± 0.93	329.67	6.79 ± 1.12	590.29	94.41 ± 0.72
F1 (50 °C)	18.86 ± 0.46	99.99	40.41 ± 0.42	199.98	31.44 ± 1.04	329.89	2.83 ± 0.06	589.17	96.81 ± 0.16
F2 (50 °C)	17.30 ± 0.29	98.49	38.31 ± 0.19	199.78	37.02 ± 2.58	329.56	4.51 ± 0.96	588.07	96.34 ± 0.59
F3 (50 °C)	14.02 ± 0.17	99.83	22.30 ± 1.97	199.91	47.58 ± 0.27	329.94	5.90 ± 0.05	590.08	93.55 ± 0.00

Where $\bar{x}$ is the arithmetic mean, SD is the standard deviation, and T (°C) is temperature

**Table 6:** Differential scanning calorimetric (DSC) thermal transitions for raw material and edible films.

Formulation	Tp (°C)	ΔH* (J/g)	°Tg (°C)	ΔH* (J/g)	°Tm (°C)	ΔH* (J/g)	°Td (°C)
F1 (60 °C)	-	-	142.73	1.29	164.74	169.94	192.00
F2 (60 °C)	-	-	140.95	1.41	155.67	19.55	159.00
F3 (60 °C)	-	-	140.02	1.08	153.43	15.18	158.00
F1 (50 °C)	-	-	138.26	1.38	154.17	27.88	165.00
F2 (50 °C)	-	-	135.86	0.64	155.31	26.40	162.00
F3 (50 °C)	-	-	138.91	1.13	155.45	28.17	164.00
Modified starch	59.60	4.04	-	-	157.00	-	160.63
Starch	59.18	4.30	-	-	156.93	-	160.95
Pulverized Nostoc	-	-	85.31	-	155.71	192.36	160.07

Tp: gelatinization temperature, Tg: temperature of transition vitrea, Tm: temperature of fusion, Td: degradation temperature y, ΔH: enthalpy variation.

**Table 7:** Mechanical properties of edible films.

Edible Film	Tensile Strength (N/mm^2^)	Elongation at Break (%)	Young’s Modulus (N/mm^2^)
	$\bar{x}$ ± SD	$\bar{x}$ ± SD	$\bar{x}$ ± SD
F1-60 °C	0.31 ± 0.11 ^c^	3.03 ± 3.06 ^b^	10.10 ± 3.06 ^d^
F2-60 °C	0.57 ± 0.00 ^b^	3.58 ± 0.16 ^b^	16.22 ± 0.69 ^cd^
F3-60 °C	4.10 ± 0.17 ^a^	4.18 ± 0.28 ^a^	97.92 ± 3.39 ^a^
F1-50 °C	0.35 ± 0.09 ^c^	3.03 ± 0.31 ^b^	11.84 ± 4.23 ^cd^
F2-50 °C	0.94 ± 0.41 ^b^	3.42 ± 0.18 ^b^	20.73 ± 1.06 ^c^
F3-50 °C	3.02 ± 0.41 ^a^	4.22 ± 0.10 ^a^	76.45 ± 5.00 ^b^

Results expressed as mean ± SD. Different letters in the same column indicate significant differences between treatments using Tukey’s test (*p* < 0.05).

## Data Availability

The original contributions presented in the study are included in the article; further inquiries can be directed to the corresponding authors.
